# Entrepreneurial well-being and performance: antecedents and mediators

**DOI:** 10.3389/fpsyg.2023.1112397

**Published:** 2023-10-19

**Authors:** Luca Tisu, Delia Vîrgă, Toon Taris

**Affiliations:** ^1^Department of Psychology, West University of Timișoara, Timișoara, Romania; ^2^Institute for Advanced Environmental Research (ICAM), West University of Timișoara, Timișoara, Romania; ^3^Social, Health and Organizational Psychology Department, Utrecht University, Utrecht, Netherlands

**Keywords:** psychological capital, well-being, performance, satisfaction, work-life balance, entrepreneurs

## Abstract

Entrepreneurial well-being is tied to increasing firm performance because entrepreneurs possess additional resources to invest in their businesses. However, research integrating antecedents, specific mechanisms related to the emergence of entrepreneurial well-being (EWB), and performance is scarce. Furthermore, the collective impact of their roles as entrepreneurs and individuals outside the work context is yet to be investigated concerning venture performance. The present study addresses these issues by presenting and testing a comprehensive model employing entrepreneurs’ psychological capital as an antecedent of EWB and, indirectly, performance. We investigate this relationship through a serial mediation mechanism enabled by work engagement and entrepreneurial satisfaction regarding entrepreneurs’ work roles. Also, we employ work-life balance and mental health as mediators regarding their home roles. Drawing on data from 217 Romanian entrepreneurs, structural equation modeling analyses supported our model. PsyCap was a precursor of entrepreneurial satisfaction both directly and through work engagement. Also, PsyCap predicted entrepreneurs’ mental health directly and through work-life balance. Furthermore, both EWB components – entrepreneurial satisfaction and mental health – were associated with business performance. Hence, our model provides valuable insights regarding the interplay between entrepreneurs’ work and home roles and their relation to EWB and venture performance. It also provides the basis for future interventions that can psychologically prepare entrepreneurs to be successful in their entrepreneurial endeavors.

## 1. Introduction

Entrepreneurs who experience psychological well-being are prone to be high performers because they possess additional psychological resources to sustain their entrepreneurial efforts (Stephan, 2018; Wiklund et al., 2019). This is linked to increased business performance (Gorgievski and Stephan, 2016; Dijkhuizen et al., 2016b), thus supporting the economic (i.e., economic growth) and societal advantages (i.e., job creation) of entrepreneurship (Van Praag and Versloot, 2007; Bosma and Kelley, 2019). Entrepreneurial well-being (hereinafter EWB) is defined as “the experience of satisfaction, positive affect, infrequent negative affect, and psychological functioning about developing, starting, growing, and running an entrepreneurial venture” (Wiklund et al., 2019, p. 582). Based on the definition proposed by Wiklund et al. (2019), at least two distinct EWB components emerge: (1) *entrepreneurial satisfaction* – an overall positive judgment regarding one’s quality of life as an entrepreneur (i.e., cognitive EWB component; Pavot and Diener, 2008), and (2) *mental health* – the presence of positive affect, infrequent negative affect, and optimal psychological functioning (i.e., affective EWB component; Keyes, 2014). Significantly, according to the Conservation of Resources (COR; Hobfoll, 2011) theory, EWB can act as a self-regulatory mechanism (Stephan, 2018), allowing entrepreneurs to draw on multiple cognitive resources (e.g., opportunity recognition, creative thinking) to invest in their business, reflecting in their business performance (Wu, 2007). Researchers have identified several precursors of EWB, such as work and personality characteristics (for synthesis, see Stephan, 2018; Kleine and Schmitt, 2021), and linked EWB components to business performance (Gorgievski and Stephan, 2016; Dijkhuizen et al., 2016b).

Nevertheless, current statistics show that almost one in five new ventures succumbs during their first year, while more than half fail to reach five years of existence (Eurostat, 2021). What factors lead to these survival rates if the necessary funding is provided and the business plans settled? Next to financial reasons, entrepreneur-dependent factors play a crucial role (DeTienne and Wennberg, 2016). From this perspective, one potential explanation is the absence of developable psychological resources that help entrepreneurs thrive as business owners (Laguna et al., 2017). The lack of psychological resources on which entrepreneurs can rely can turn positive entrepreneurial activities into stressors (Tisu and Vîrgă, 2021), leading to lower levels of entrepreneurial satisfaction (i.e., cognitive EWB component) and the decision to exit their business. A second potential explanation is a collision between entrepreneurs’ professional and personal lives. Entrepreneurship is a highly resource-demanding career path, leading to stress and straining entrepreneurs’ mental health (i.e., affective EWB component) (Andersson, 2008). As such, entrepreneurs could decide to exit their entrepreneurial role to safeguard their social life (DeTienne and Wennberg, 2016). In this paper, we argue that *entrepreneurs’ psychological capital* – a success-oriented mindset comprising self-efficacy, hope, resilience, and optimism (hereinafter PsyCap; Luthans et al., 2007) represents a vital, developable psychological resource (Grover et al., 2018) that helps entrepreneurs (1) handle their professional and personal roles efficiently, (2) draw resources from both roles to fully experience EWB, and (3) potentially be linked to enhanced business performance via EWB.

Specifically, we propose entrepreneurs’ PsyCap as a distal precursor of business performance (i.e., financial business growth; Dijkhuizen et al., 2018) through a serial mediation mechanism comprising both entrepreneurs’ professional and personal lives. Aligned with the propositions of the Job-Demands Resources (JD-R; Bakker and Demerouti, 2017) theory, we argue that PsyCap equips entrepreneurs with the necessary mindset to become more engaged in their role (i.e., work engagement) while also being able to strike a better balance between their professional and personal lives (i.e., work-life balance; Kalliath and Brough, 2008). Through these mediating mechanisms (i.e., work engagement and work-life balance), we expect PsyCap to be linked to cognitive and affective EWB. First, we expect entrepreneurs who experience work engagement – a high-energy activating state comprising vigor, dedication, and absorption (Schaufeli et al., 2002) also to form overall favorable judgments regarding their lives as entrepreneurs (i.e., entrepreneurial satisfaction) due to the positive, motivational state elicited by their engagement (Dijkhuizen et al., 2018). Second, we argue that entrepreneurs who experience work-life balance can shield their mental health, thus experiencing affective EWB. As such, we propose a comprehensive yet parsimonious model integrating one developable psychological resource (i.e., PsyCap) as an antecedent, two domain-specific explanatory mechanisms (i.e., work engagement and work-life balance), and two EWB sources (i.e., entrepreneurial satisfaction and mental health), to test the assumption that person-dependent EWB factors are associated with business performance.

This study covers two significant gaps in knowledge. First, we integrate both entrepreneurs’ life avenues (i.e., professional and personal life) into a unitary model to investigate how they jointly sustain EWB. As Stephan (2018) notes, entrepreneurs’ work and private roles are closely intertwined and are expected to impact EWB and performance together. While entrepreneurial activities can elicit positive evaluations due to attaining work-related goals (e.g., business growth; Dijkhuizen et al., 2016a,b), this sustained effort can also be linked to a decrease in mental health due to rising stress levels (Andersson, 2008; Wincent et al., 2008; Cardon and Patel, 2015; Lerman et al., 2020). Entrepreneurs could, however, protect their mental health and, subsequently, affective EWB by engaging in various activities in their home role, such as spending time with family and friends, which can reduce stress levels and replenish their resource reservoir (Kinnunen et al., 2011; Rodríguez-Muñoz et al., 2014). This should enable entrepreneurs to maintain (1) good mental health through their home role via work-life balance (i.e., affective EWB) and (2) entrepreneurial satisfaction through their work via work engagement (i.e., cognitive EWB).

**Figure 1:**
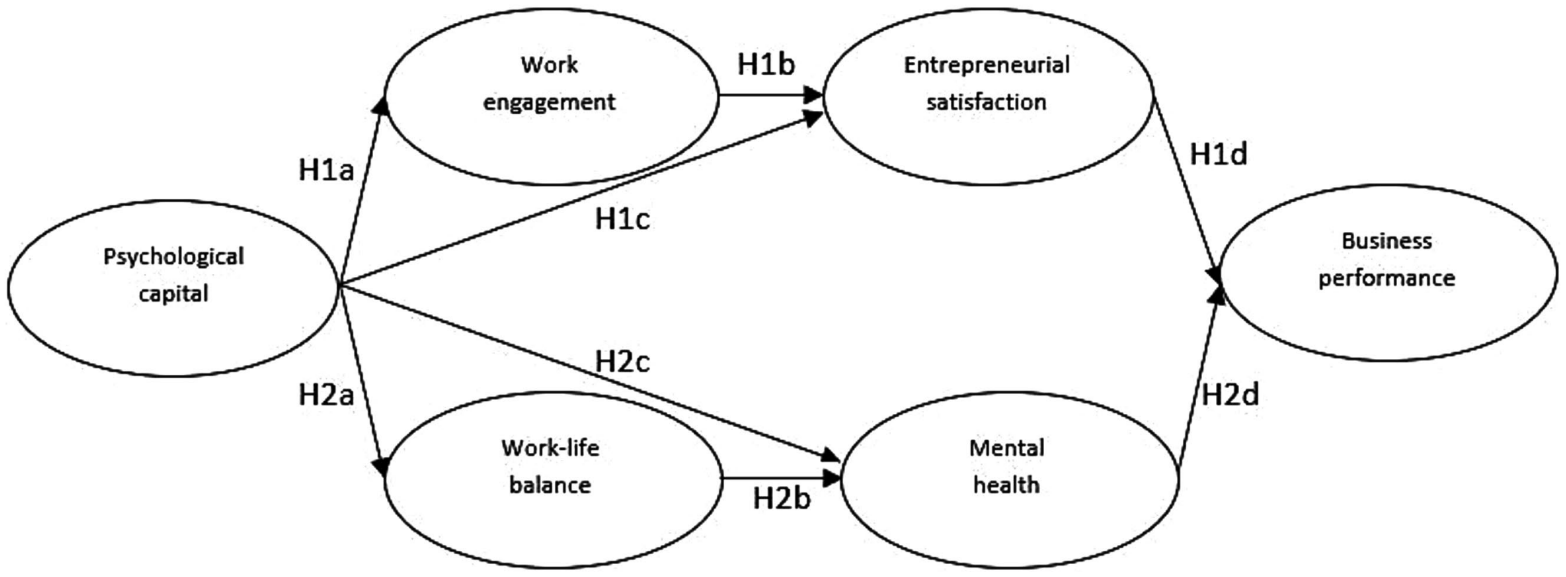
The hypothesized model.

Second, by investigating positive precursors (i.e., PsyCap) and explanatory mechanisms (i.e., work engagement and work-life balance) associated with the emergence of EWB, we seek to link EWB to business performance in a unitary model that includes antecedents and mediators. Stephan (2018) proposes that EWB acts as a self-regulatory mechanism in entrepreneurial activities. This assumption is based on COR theory, which states that individuals possessing sufficient resources (i.e., PsyCap, EWB) are inclined to invest their existing bundle of resources to orchestrate additional resource gains (Hobfoll, 2011). This assumption is also doubled by the Broaden-and-Build theory (Fredrickson, 2001), according to which the accumulation of resources, such as EWB, allows individuals to broaden their behavioral, emotional, and cognitive repertoire. Hence, entrepreneurs who are satisfied with their lives as entrepreneurs and exhibit good mental health due to the mechanisms mentioned above (i.e., PsyCap, work engagement, work-life balance) can devote further energy to their work (Hobfoll, 2011). Therefore, by experiencing EWB, entrepreneurs should free cognitive mechanisms (e.g., creative thinking, opportunity recognition), allowing them to engage in behaviors beneficial for their business (e.g., developing a new product, attracting new investors), reflecting on their business performance (Wu, 2007). Thus, the proposed model can present entrepreneurs, practitioners, and policymakers with valuable insights regarding promoting EWB from a two-pronged perspective, allowing entrepreneurs to invest more resources into their ventures and possibly be linked to enhanced business performance. For an overview of the proposed model, for which we provide the theoretical underpinnings in the following paragraphs, please see Figure 1.

### 1.1. Entrepreneurs’ PsyCap – an antecedent of EWB

In this study, we employ entrepreneurs’ PsyCap (Luthans et al., 2007) as a common antecedent of both EWB components – entrepreneurial satisfaction and mental health. PsyCap is a malleable, personal resource (i.e., developable cognitive-emotional states that facilitate goal attainment; Van den Heuvel et al., 2010) that equips individuals with a success-oriented mindset. According to the JD-R theory (Bakker and Demerouti, 2017), possessing such personal resources triggers a motivational mechanism that allows individuals to become more energetic and invest more effort into their activities, reflecting in positive work-related (i.e., job satisfaction; Newman et al., 2014) and home-related (i.e., mental health; Luthans et al., 2013) outcomes. Being a state-like, higher-order construct, PsyCap comprises four components: self-efficacy (confidence to take on and succeed in challenging tasks), optimism (expecting to succeed now and in the future), hope (persisting toward goals and, if necessary, rethinking paths for successful goal attainment), and resilience (when facing problems and adversity, sustaining and bouncing back to attain success) (Luthans et al., 2007). Exploring PsyCap as a unitary construct rather than taking each component separately permits an investigation closer to reality. Based on the tenets of COR theory, resources tend to travel together in the so-called resource caravan passageways (Hobfoll, 2011). Individuals with high levels of self-efficacy are also likely to possess resilience and hope (Luthans et al., 2007). Thus, aligned with past research (Luthans et al., 2007; Siu, 2013; Grover et al., 2018), capturing these resources into a single, super-ordinate construct (i.e., PsyCap) is recommended.

Next, to these theoretical considerations, we have selected this specific construct for two other reasons. First, PsyCap is not a domain-specific resource being linked to both work and private life outcomes (Luthans et al., 2007, 2013). Thus, it can fuel positive outcomes in entrepreneurs’ work roles, allowing them to experience entrepreneurial satisfaction while also enabling them to sustain their mental health through the home role. Indeed, previous studies have already linked PsyCap (Baron et al., 2016; Bockorny and Youssef-Morgan, 2019) or its components (i.e., self-efficacy; Laguna et al., 2017) to EWB outcomes in entrepreneurs’ work roles. Luthans et al. (2013) demonstrate that it can also lead to positive home role outcomes (e.g., spending more time with the family). Therefore, we expect PsyCap to be a resource that allows entrepreneurs to handle both roles effectively, enabling the emergence of EWB. Second, PsyCap is developable through interventions (for synthesis, see Lupșa and Vîrgă, 2018). This is particularly important because, as Obschonka and Stuetzer (2017) argue, to ensure successful interventions in entrepreneurship, scholars must first identify relevant malleable psychological resources that can be fostered through interventions. We will now provide a more in-depth theoretical argumentation regarding the link between PsyCap and EWB based on the two life avenues (work and home) this study explores.

### 1.2. PsyCap, entrepreneurial well-being, and performance: the work-life avenue

Possessing personal resources, such as PsyCap, allows individuals to draw upon those resources to devote more energy to their work (Van den Heuvel et al., 2010; Youssef-Morgan and Luthans, 2015; Bakker and Demerouti, 2017). That is also the case for entrepreneurs (Baron et al., 2016; Bockorny and Youssef-Morgan, 2019). Approaching work with an extended resource reservoir facilitates more efficient task completion and goal attainment (Bakker and Demerouti, 2017). In turn, being able to meet one’s standards and objectives elicits a sense of satisfaction (Youssef-Morgan and Luthans, 2015), a component of EWB, and a precursor of business performance (Dijkhuizen et al., 2016b; Stephan, 2018). Therefore, PsyCap should fuel the emergence of EWB, with the latter enabling entrepreneurs to devote more energy to their work, thus helping their businesses grow and thrive.

However, PsyCap is a psychological resource that requires various mechanisms to be translated into positive outcomes (Cenciotti et al., 2017; Tisu et al., 2020). Researchers demonstrated that while possessing PsyCap is a prerequisite for being satisfied (Luthans et al., 2007; Newman et al., 2014) and performing well (Tisu et al., 2020), these links occur either through a motivational component (i.e., work engagement; Tisu et al., 2020) or through a behavioral one (i.e., job crafting; Cenciotti et al., 2017). For instance, while being resilient is essential when encountering a setback, one must draw energies from this resource and start over enthusiastically to achieve one’s objectives. Indeed, the JD-R theory also stipulates that the link between personal resources and positive outcomes is mediated by work engagement (Bakker and Demerouti, 2017). Based on these arguments, we employ work engagement as an explanatory mechanism that links PsyCap to entrepreneurial satisfaction.

Work engagement is “a positive, fulfilling, work-related state of mind that is characterized by vigor (feeling energized and willing to invest effort), dedication (being involved and experiencing a sense of enthusiasm), and absorption (being fully concentrated and happily engrossed in one’s work)” (Schaufeli et al., 2002, p. 74). Entrepreneurs usually report high levels of work engagement due to the nature of their jobs (Dijkhuizen et al., 2016b; Laguna et al., 2017). Entrepreneurs carry out many activities in their work, from meeting with clients or handling paperwork to devising new products. While being involved in these activities is expected to generate work engagement because entrepreneurs find them captivating and fulfilling, it is unlikely that all activities generate such positive states. Thus, as a high-energy activating mechanism, work engagement is expected to fluctuate based on the activity entrepreneurs are involved in (Dijkhuizen et al., 2016b). However, should the aggregate of these positive experiences (e.g., meetings with clients) exceed those that do not elicit work engagement (e.g., handling paperwork), this can allow the emergence of a more stable indicator of work well-being, namely entrepreneurial satisfaction (Dijkhuizen et al., 2018).

Therefore, possessing relevant psychological resources to draw upon in their activities (i.e., PsyCap) can enable entrepreneurs to find more of their tasks enjoyable and engaging (i.e., work engagement). This, in turn, should lead to the emergence of entrepreneurial satisfaction, the durable, cognitive, work-related component of EWB, a precursor of business performance (Stephan, 2018).

#### 1.2.1. Entrepreneurs’ PsyCap and work engagement

Existing findings have already linked PsyCap to EWB. For instance, Baron et al. (2016) and Bockorny and Youssef-Morgan (2019) found an association between PsyCap and entrepreneurs’ subjective well-being. Furthermore, Van den Heuvel et al. (2010) argue that the four constructs shaping PsyCap represent a powerful predictor of the interrelated components of work engagement, especially in the face of uncertainty, a central aspect of entrepreneurs’ work-life (Yang et al., 2017). Hence, entrepreneurs who have the confidence to engage in challenging work, plan and find alternative routes to achieve their goals, and quickly recover after setbacks should be prone to cope with demands arising from their activities. Therefore, they can find joy, invest more effort in various tasks, and experience more of their work-related activities as fulfilling. Thus, we hypothesize that:

Hypothesis 1a: PsyCap is positively associated with entrepreneurs’ work engagement.

#### 1.2.2. Entrepreneurs’ work engagement and entrepreneurial satisfaction

Not all entrepreneurial activities are bound to generate work engagement. However, should the aggregate of the positive activities surpass the negative ones, individuals can experience entrepreneurial satisfaction. Although in past studies, entrepreneurial satisfaction has also been conceptualized based on indicators regarding how satisfied entrepreneurs are with their businesses, such as income or relation with stakeholders (Schjoedt, 2009; Song and Guo, 2020), scholars have recently argued that this approach reflects rather entrepreneurial performance/success than entrepreneurial satisfaction (Gorgievski et al., 2014; Dijkhuizen et al., 2018; Tisu and Vîrgă, 2022). Thus, in this study, we follow Pavot and Diener’s (2008) perspective on satisfaction – a cognitive overall positive evaluation of one’s quality of life (i.e., a cognitive form of well-being), and use it to capture entrepreneurs’ assessment of the quality of their lives as entrepreneurs. Thus, in this study, entrepreneurial satisfaction reflects the cognitive side of EWB, encompassing perceptions and evaluations regarding the quality of life as an entrepreneur. As mentioned, experiencing a positive motivational state through high-energy activating aspects, such as being invested in one’s work, experiencing a state of enthusiasm regarding one’s tasks, and feeling energized by most entrepreneurial activities (i.e., work engagement), can also be associated with a more passive yet continuous form of well-being, namely entrepreneurial satisfaction. Both theory and existing evidence support this assumption. According to the JD-R theory, work engagement acts as a precursor of positive work outcomes, such as satisfaction with one’s job (Newman et al., 2014). Furthermore, Dijkhuizen et al. (2016b) identify an association between work engagement and domain-specific entrepreneurial satisfaction (i.e., job satisfaction), with the latter being more strongly related to performance. Their findings support the claim that perceiving most entrepreneurial activities as engaging is linked to higher satisfaction with their lives as entrepreneurs. This leads us to expect that:

Hypothesis 1b: Work engagement is positively associated with entrepreneurial satisfaction.

#### 1.2.3. Entrepreneurs’ PsyCap and entrepreneurial satisfaction

PsyCap allows entrepreneurs to face their activities with a success-oriented mindset. Self-efficacy equips them with the confidence to take on challenging tasks, such as approaching potential investors. Hope can enable them to set new goals for their business (e.g., devise new products/services), and persevere in following and finding alternative pathways to achieve them. Optimism allows them to generate a positive perspective regarding various business-related outcomes (e.g., reaching business objectives), while resilience should enable them to recover after potential setbacks. Altogether, these aspects can facilitate goal attainment, an essential component of satisfaction (Judge and Ilies, 2004; Sim and Lui, 2020). Indeed, previous studies have linked PsyCap to satisfaction in samples of employees (Karatepe and Karadas, 2015) and entrepreneurs (Baron et al., 2016; Bockorny and Youssef-Morgan, 2019). Hence, entrepreneurs who attain their goals by employing their PsyCap should experience content and fulfillment regarding their lives as entrepreneurs. Considering these arguments, we expect that:

Hypothesis 1c: PsyCap is positively associated with entrepreneurial satisfaction.

#### 1.2.4. Entrepreneurial satisfaction and performance

Business performance is a complex, multidimensional construct that has been operationalized based either on financial (e.g., revenue growth), non-financial indicators (e.g., business reputation), or a combination of the two (Rauch et al., 2009). In this study, we adopt a financial business growth perspective as an indicator of business performance, as proposed by Stephan and Richter (2006). This approach aligns with past research, which argues that if a business shows an increase in revenue, turnover, and/or number of employees, this reflects a prosperous and performant business (Dijkhuizen et al., 2016b, 2018). Considering that business owners are key agents in the growth and development of the business (Baron, 2007; Stephan, 2018), they need to invest multiple resources in their role (e.g., time and energy) to enhance business performance. By experiencing entrepreneurial satisfaction, entrepreneurs will possess and be more inclined to invest additional resources into their business-related activities, reflecting on business performance. For instance, they might devote additional resources to devise or perfect products/services, resulting in the firms’ financial expansion. This assumption is rooted in COR theory (Hobfoll, 2011) and supported by the existing evidence (Dijkhuizen et al., 2016b; Stephan, 2018). Based on the COR theory, those who possess sufficient resources should be able to orchestrate additional resource gains (Hobfoll, 2011). Thus, entrepreneurial satisfaction represents a vital psychological resource that enables entrepreneurs to devote further energies to their business, acting as a self-regulatory mechanism. Specifically, entrepreneurial satisfaction should allow entrepreneurs to draw on multiple cognitive resources (e.g., opportunity recognition and creative thinking) to invest in their business (Zhang, 2014; Stephan, 2018), resulting in enhanced business performance (Dijkhuizen et al., 2016b). Thus, we propose:

Hypothesis 1d: Entrepreneurial satisfaction is positively associated with business performance.

### 1.3. PsyCap, entrepreneurial well-being, and performance: the personal-life avenue

PsyCap is a psychological resource linked not only to work-related outcomes but also to the home role of individuals (Luthans et al., 2013; Youssef-Morgan and Luthans, 2015). Research has demonstrated that individuals can rely on their PsyCap to achieve objectives such as spending more time with others and reporting good mental health (Luthans et al., 2013). Therefore, we expect PsyCap to be related to entrepreneurs’ mental health, the affective EWB component derived from their private role as individuals. We have chosen to place mental health as stemming from this life avenue because, as research shows, entrepreneurs report high levels of stress in their work role (Wincent et al., 2008; Cardon and Patel, 2015; Lerman et al., 2020), which is bound to affect their mental health. Therefore, we argue that the home role of entrepreneurs ought to compensate for and protect their mental health. In this paper, we conceptualize mental health as the presence of positive affect, infrequent negative affect, and optimal psychological functioning (Keyes, 2014). As this hedonic-derived definition implies, entrepreneurs’ mental health does not reflect an absence of illness but rather the presence of positive feelings (e.g., joy) and sparse negative feelings (e.g., stress), allowing individuals to function optimally. Based on this conceptualization and aligned with Pavot and Diener’s (2008) distinction between affective and cognitive well-being, mental health reflects the affective side of EWB in this study.

The present investigation places work-life balance as a mediator between PsyCap and mental health regarding entrepreneurs’ home roles. Work-life balance represents “the individual perception that work and non-work activities are compatible and promote growth in accordance with an individual’s current life priorities” (Kalliath and Brough, 2008, p. 326). Existing studies indicate that entrepreneurs encounter difficulties in attaining work-life balance and may sacrifice their personal lives to achieve work-related objectives (Ezzedeen and Zikic, 2017; Adisa et al., 2019). This approach can take its toll on entrepreneurs’ mental health because they will lack relevant resources such as social support (Stephan, 2018) or a feeling of relatedness (Shir et al., 2019), which are necessary for psychological well-being (Ryan and Deci, 2017). Hence, it is vital that entrepreneurs also engage in activities with family and friends, such as a night out, that can generate social support, replenish entrepreneurs’ resource pool, and reduce stress levels (Kinnunen et al., 2011; Rodríguez-Muñoz et al., 2014), thus protecting their mental health. Alternatively, they may even discuss their business-related ruminations with fellow entrepreneurs, thus generating a feeling of relatedness (Shir et al., 2019) and, as such, experiencing a state of psychological well-being. However, being immersed in one’s job extensively can leave entrepreneurs with no resources to engage in activities like those described above. Nevertheless, PsyCap could enable them to set specific non-work-related goals (e.g., spending time with friends) and provide the cognitive resources to attain them (Luthans et al., 2013).

Considering this, PsyCap can help entrepreneurs balance work and non-work activities. This can allow them to gain social support or a feeling of relatedness and reduce stress levels, thus protecting and enhancing entrepreneurs’ mental health. In turn, generating the second EWB component (i.e., mental health) should also reflect on business performance (Stephan, 2018).

#### 1.3.1. Entrepreneurs’ PsyCap and work-life balance

PsyCap can represent a prerequisite for the emergence of work-life balance from at least two perspectives – (1) a work-related resource-gleaning perspective and (2) a home-related resource investment perspective, both rooted in the COR theory (Hobfoll, 2011). Regarding the first approach, PsyCap equips entrepreneurs with the necessary cognitive and emotional resources to achieve work-related goals (Van den Heuvel et al., 2010). Having a clear set of work-related goals and being able to find alternative routes of attaining them (hope), combined with the perseverance to achieve these (self-efficacy and resilience), should enable entrepreneurs to be more efficient at work (Kautonen et al., 2015). This would allow entrepreneurs to glean resources (e.g., time) in their work role and possibly invest these in complementary non-work activities (e.g., spending time with family and friends). However, some entrepreneurs see their work as a life priority (Ezzedeen and Zikic, 2017; Adisa et al., 2019) and thus may decide to reinvest the gleaned resources into their work role rather than their home role. Nevertheless, as Luthans et al. (2013) and Siu (2013) demonstrate, PsyCap is a resource that helps individuals set and achieve family-related goals. Accordingly, entrepreneurs can employ their PsyCap and draw upon it to schedule and engage in non-work-related activities, such as a night out with friends, doing volunteer work, practicing sports, or any other activity that helps them replenish their resource reservoir (Kinnunen et al., 2011; Rodríguez-Muñoz et al., 2014). Based on these arguments, we argue that:

Hypothesis 2a: PsyCap is positively associated with entrepreneurs’ work-life balance.

#### 1.3.2. Entrepreneurs’ work-life balance and mental health

Entrepreneurs who cannot balance work and home-related experience increased work-home conflict (Nguyen and Sawang, 2016). To reduce conflict, some entrepreneurs sacrifice their private lives and entirely focus on their jobs (Ezzedeen and Zikic, 2017; Adisa et al., 2019). This can prove problematic for entrepreneurs’ mental health because non-work-related interactions satisfy entrepreneurs’ need for relatedness (Shir et al., 2019), generate social support (Stephan, 2018), and protect their optimal psychological functioning (Ryan and Deci, 2017). Current evidence suggests that work-life balance is linked to optimal mental health (Haar et al., 2014). While Haar et al.’s (2014) work focused on employees across different nations, we expect to find a similar link between work-life balance and mental health among entrepreneurs. Being able to juggle work and home role activities should enable entrepreneurs to engage in various activities they find enjoyable outside of work. As such, they could replenish their resource pool and reduce stress levels (i.e., decreased negative affect) (Kinnunen et al., 2011). It would also allow them to experience a sense of kinship and joy through non-work-related activities (i.e., presence of positive affect) rather than experiencing strain due to conflict between competing roles. Hence, attaining a work-life balance would reduce entrepreneurs’ mental health strain, enabling optimal psychological functioning. Accordingly, we hypothesize that:

Hypothesis 2b: Work-life balance is positively associated with entrepreneurs’ mental health.

#### 1.3.3. Entrepreneurs’ PsyCap and mental health

PsyCap is an antecedent of mental health (Luthans et al., 2013). For instance, Estiri et al. (2016) found a positive relationship between Iranian nurses’ PsyCap and their mental health. Furthermore, Krasikova et al. (2015) obtained similar results on a robust sample of US soldiers. Indeed, having an optimistic, success-oriented mindset provides cognitive agentic mechanisms that allow individuals to maintain their mental health, and we expect entrepreneurs to make no exception. First, the combined effect of the PsyCap components acts as a buffer in experiencing high-stress levels (Baron et al., 2016). Especially the optimistic outlook on life and the resilience to recover quickly after setbacks should positively impact entrepreneurs’ mental health. Second, pursuing meaningful yet challenging goals (hope), supported by the belief that these goals may be obtained (self-efficacy), should enable entrepreneurs to surmount possible feelings of anxiety or other negative ruminations. This is also bound to have a positive impact on their mental health. Thus, we assume that:

Hypothesis 2c: PsyCap is positively associated with entrepreneurs’ mental health.

#### 1.3.4. Entrepreneurs’ mental health and business performance

While entrepreneurial satisfaction allows entrepreneurs to experience EWB from a cognitive perspective, their mental health leads to decreased negative affect and optimal psychological functioning, reflecting on affective EWB. Taken together, they enable entrepreneurs to experience enhanced EWB (Wiklund et al., 2019), a precursor of business performance (Stephan, 2018). Since experiencing good mental health is negatively associated with resource-draining stressors, such as anxiety, stress, or negative ruminations (Berwick et al., 1991), entrepreneurs who exhibit good mental health should have more resources. Therefore, we expect entrepreneurs with good mental health to possess additional emotional resources (e.g., positive affect), complementing the cognitive resources garnered by entrepreneurial satisfaction (e.g., creative thinking). They may invest these in enhancing their business performance, as hypothesized by COR theory (Hobfoll, 2011). Hence, our final hypothesis is:

Hypothesis 2d: Entrepreneurs’ mental health is positively associated with business performance.

### 1.4. Alternative hypothetical models

Up to this point, we have argued that PsyCap is a precursor of entrepreneurs thriving in both their work and home roles and will, as such, show an association with business performance. To generate a precise and parsimonious model of antecedents and mediators of EWB occurrence, we did not include crossover relationships between the two avenues. However, current findings indicate that work engagement is an antecedent of mental health (Shimazu et al., 2018), while other studies consider work-life balance as a precursor of job satisfaction (Haar et al., 2014). Considering that the two roles of entrepreneurs are closely connected (Stephan, 2018), it is possible to find relationships that cross from the work role of entrepreneurs to their private life and vice versa. Consequently, we propose a series of alternative models where the employed mediators impact both roles entrepreneurs engage in, thus extending our initial hypothetical model. Hence, the first alternative model posits a crossover relationship where work engagement predicts both entrepreneurial satisfaction (hypothesized) and entrepreneurs’ mental health. The second alternative model investigates whether work-life balance positively impacts mental health (hypothesized) and entrepreneurial satisfaction. Finally, our third alternative model combines all previously mentioned relationships. Specifically, in this model, we employ both primary mediators (i.e., work engagement and work-life balance) as a precursor of both EWB components (i.e., entrepreneurial satisfaction and mental health).

## 2. Methods

### 2.1. Participants and procedure

All participants had to meet the inclusion criteria proposed by Baron (2007), who defined entrepreneurs as individuals who are simultaneously (1) founders, (2) owners, and (3) managers of their firms. We chose these three indicators as inclusion criteria because founders exhibit entrepreneurial orientation as well, a critical component of entrepreneurship, as opposed to next-generation owners who were not involved in the creation of the business (Ljungkvist et al., 2019). Additionally, respondents also had to be the current managers of their business to ensure that they are still actively involved in managing their venture and not passive recipients of revenue from a business they are no longer leading. When collecting the data, we used a snowball sampling procedure (Babbie, 2013). The researchers contacted a network of entrepreneurs from Western Romania and invited them to complete the questionnaire. Respondents were then asked to provide contact details of two other entrepreneurs they knew whom the researchers could recruit as additional participants in the study. This approach resulted in 411 contacted entrepreneurs, of which 217 returned the completed questionnaire (a 53% response rate). Respondents were contacted by the researchers and received weekly reminders to complete the questionnaire during the data collection phase (1 month). All participants were informed about the research scope, the confidentiality of the data, and their right to retreat from the study at any moment. Participation was, therefore, voluntary, and no incentives were offered for their participation.

More than half of the participants (61.2%) were males; the mean age was 39.4 years (SD = 11.04); 65% were married, and 71% held at least a Bachelor’s degree. Their businesses ranged from import–export and construction to food services and IT/high-tech. Participants had, on average, 16.7 years (SD = 10.68) of work experience, with a mean total entrepreneurial experience of 9.77 years (SD = 7.61). The mean tenure in leading their current firm was 8.24 years (SD = 7.33), with most organizations (73%) reporting having an annual profit of less than €50.000 and 14% reporting a yearly profit of €100.000 or more.

### 2.2. Instruments

For most instruments, we relied upon tried-and-tested Romanian versions of existing international instruments (i.e., PsyCap, work engagement, and mental health; Tisu et al., 2020). The other instruments were adapted using the standard back-translation technique (Brislin, 1970).

Psychological capital was measured with the 24-item PsyCap Questionnaire (Luthans et al., 2007; Lupșa and Vîrgă, 2018). The questionnaire comprises four subscales, each with six items, and was adapted to reflect aspects of the activity as an entrepreneur: self-efficacy (“I feel confident presenting information to a group of stakeholders (clients, investors).”), resilience (“I usually take stressful things in stride in my work as an entrepreneur.”), hope (“There are lots of ways around any problem in my activity as an entrepreneur.”), and optimism (“I approach my activity as an entrepreneur as if every cloud has a silver lining.”). The items were evaluated on a six-point Likert scale (1 = *“strongly disagree”*, 6 = *“strongly agree”*).

Work engagement was measured with the Utrecht Work Engagement Scale (UWES-9; Schaufeli et al., 2006; Vîrgă et al., 2009). The scale consists of three dimensions, and each is measured with three items: vigor (“When I get up in the morning, I feel like going to work.”), dedication (“I find the work that I do full of meaning and purpose.”), and absorption (“Time flies when I am working.”) (0 = *“never”*, 6 = *“always”*).

Entrepreneurial satisfaction was measured based on the adapted version of the Satisfaction with Life Scale (SWLS; Diener et al., 1985), as previously done by Dijkhuizen et al. (2018). The scale consists of five items, and answers were registered on a 5-point scale (1 = *“totally disagree”*, 5 = *“totally agree”*). A sample item is: “I am satisfied with my life as an entrepreneur.”

Work-life balance was measured with the four-item scale developed by Brough et al. (2014). A sample item is: “Overall, I believe that my work and non-work life are balanced.” (1 = *“strongly disagree”*, 5 = *“strongly agree”*).

Mental health was measured with a five-item mental health screening test (Berwick et al., 1991). This scale initially measured the presence of mental health complaints and included two items tapping the absence of such complaints. The scores on the other three items were reversed to obtain a positive mental health score. A sample item is: “During the past month, how much of the time have you felt calm and peaceful?” (1 = *“never”, 6 = “always”*).

Business performance was measured using self-reported business growth indicators. We employed three items referring to business growth during the last fiscal year in terms of profit, business turnover, and number of employees (Stephan and Richter, 2006). A sample item is: “How did the profit of the company change over the past twelve months?” (1 = *“has declined”*, 5 = *“has grown”*).

Most of Cronbach’s alpha values were above the cut-off values indicated by Schmitt (1996) of 0.70. Resilience (0.67) and optimism (0.64) made an exception (as presented in Table 1). However, as Nunnally (1994) mentions, values in the range of 0.60 are also acceptable. Details regarding item wording, descriptive statistics for individual items, as well as factor loadings, and results of confirmatory factor analyses (CFA) for each scale can be viewed in Supplementary Table 1 (Supplementary material). In short, all items are normally distributed, all factor loadings surpass the minimum 0.40 threshold (Tabachnick and Fidell, 2007), and the individual CFAs yield good fit indices (Marsh et al., 2005).

**Table 1:** Means, standard deviations, correlation coefficients, and reliability coefficients table.

Observed Variables	*M*	*SD*	AVE	1	2	3	4	5	6	7	8	9	10	11	12	13	14	15	16	17
1. Age	38.82	11.37	–	–																
2. Gender	1.6	0.50	–	0.01	–															
3. Entrepreneurial experience	9.31	7.79	–	0.66**	0.14*	–														
4. Tenure current business	8.05	7.15	–	0.47**	0.05	0.68**	–													
5. Self-efficacy	5.15	0.69	0.90	−0.01	0.11	0.10	−0.06	(0.85)												
6. Hope	4.85	0.66	0.87	0.03	0.04	0.10	0.02	0.62**	(0.75)											
7. Resilience	4.71	0.59	0.79	0.05	0.04	0.05	−0.04	0.51**	0.61**	(0.67)										
8. Optimism	4.67	0.61	0.80	0.08	0.07	0.06	−0.04	0.48**	0.57**	0.53**	(0.64)									
9. PsyCap	4.85	0.52	0.86	0.04	0.08	0.10	−0.03	0.81**	0.86**	0.80**	0.78**	(0.89)								
10. Work-Life Balance	13.99	3.33	0.92	0.13*	−0.09	0.00	0.07	0.14*	0.17*	0.28**	0.26**	0.26**	(0.83)							
11. Mental Health	22.53	4.15	0.79	0.24**	0.01	0.09	0.09	0.30**	0.34**	0.32**	0.44**	0.43**	0.41**	(0.78)						
12. Vigor	16.95	3.38	0.78	0.13	−0.04	0.10	0.03	0.40**	0.40**	0.28**	0.30**	0.43**	−0.02	0.20*	(0.74)					
13. Dedication	16.79	3.69	0.89	0.08	−0.03	0.03	0.04	0.31**	0.37**	0.28**	0.28**	0.38**	0.01	0.30**	0.79**	(0.72)				
14. Absorption	17.09	3.34	0.78	0.05	−0.02	0.08	0.08	0.27**	0.31**	0.23**	0.25**	0.33**	−0.04	0.16*	0.75**	0.79**	(0.75)			
15. Work Engagement	50.83	9.61	0.82	0.09	−0.03	0.07	0.08	0.35**	0.39**	0.28**	0.30**	0.41**	−0.02	0.24**	0.92**	0.93**	0.92**	(0.91)		
16. Entrepreneurial satisfaction	26.87	5.25	0.91	0.06	0.05	0.04	0.06	0.27**	0.47**	0.19**	0.32**	0.39**	0.16*	0.28**	0.36**	0.36**	0.32**	0.38**	(0.92)	
17. Business performance	10.08	2.27	0.91	−0.18**	0.10	−0.20**	−0.12	0.20**	0.28**	0.13*	0.22**	0.26**	0.15*	0.23**	0.20**	0.24**	0.16*	0.22**	0.45**	(0.79)

^*p < 0.05; **p < 0.001 (two-tailed); *N* = 217; Cronbach’s α coefficients are displayed on the main diagonal. M = mean, SD = standard deviation, AVE = average variance extracted.^

### 2.3. Data analyses

Considering the difficulty in obtaining high response rates from samples of entrepreneurs (*cf.* Taris et al., 2008), we relied upon item parceling to obtain a satisfactory indicator-to-sample size ratio (Schreiber et al., 2006). Specifically, we relied upon item parceling to create factor scores as indicators for satisfaction with life, work-life balance, and mental health latent variables. Factors were created by ranking and computing the observed variables based on the factorial algorithm proposed by Rogers and Schmitt (2004). Although the rule of thumb is to generate at least three parcels per scale (Rogers and Schmitt, 2004), we were forced to create only two parcels per latent variable since our measures consisted of only four to five items. Furthermore, PsyCap and work engagement were also employed as latent variables, each consisting of its specific components: self-efficacy, hope, resilience, and hope (for PsyCap), respectively vigor, dedication, and absorption (for work engagement). Details regarding the specific allocation of items to parcels can be found in Supplementary Table 1 (Supplementary material).

The data were then analyzed using covariance-based structural modeling techniques (CB-SEM) using the lavaan package (Rossell, 2012) in R software (R Core Team, 2020). All variables included in the analyses were normally distributed, with all skewness and kurtosis values being lower than 1. We assessed the measurement and structural models using a latent variables approach (Schreiber et al., 2006; Little, 2013). Model fit was evaluated using maximum likelihood estimation; we calculated three absolute fit indices (the chi-square statistic; RMSEA – the root mean square error of approximation, and SRMR – the standardized root mean square residual) and two relative fit indices (CFI – Comparative fit index; and TLI – Tucker-Lewis index). Marsh et al. (2005) indicate that values of 0.90 or 0.95 for CFI and TLI and values of 0.08 or 0.06 represent acceptably, respectively, excellent fit indices.

First, we used CFA to test six measurement models (MM): MM1 – a model with six super-ordinate factors (PsyCap, work engagement, entrepreneurial satisfaction, work-life balance, mental health, and performance), MM2 – a model with five super-ordinate factors (entrepreneurial satisfaction and mental health were merged into an EWB factor, all other variables as individual factors), MM3 – a model with four super-ordinate factors (entrepreneurial satisfaction, mental health, and work engagement were merged into a single well-being factor, PsyCap, work-life balance, performance), MM4 – a model with three super-ordinate factors (work-life balance, performance, all other variables loading on one factor), MM5 – a model with two super-ordinate factors (performance, all other variables loading on one factor), and MM6 – a single-factor model (Podsakoff et al., 2012). As depicted in Table 2, the first model (MM1) had acceptable fit indices (*χ*^2^ (237) = 501.98, *p* < 0.001, CFI = 0.91; TLI = 0.90; RMSEA = 0.07, 90% CI [0.06, 0.08], SRMR = 0.07) with all other measurement models displaying inadequate fit factors. The chi-square difference test indicated that MM1 fitted the data better than any other measurement model (MM2 – MM6; see Table 2). Therefore, common method bias does not seem sufficient to account for the associations among the study variables. Furthermore, because MM2, where entrepreneurial satisfaction and mental health were merged into a single super-ordinate EWB factor, showed poorer fit indices than MM1, this demonstrates that while the two EWB components are related, they reflect distinct dimensions of EWB (i.e., cognitive and affective component). Additionally, we conducted another two CFAs to test the second-order, four-dimension structure of the PsyCap scale (i.e., items load on specific dimensions – self-efficacy, hope, resilience, optimism, and the four factors load on a second-order construct, reflecting PsyCap; MMff), which displayed good psychometric properties compared to a one-dimension model (i.e., all items load on a one-dimensional construct; MMof; see Table 2), demonstrating that it reflects a second-order construct.

**Table 2:** Fit statistics and model comparison for the measurement and structural models.

Model	*χ*^2^	*Df*	*χ*^2^*/df*	CFI	TLI	RMSEA [90% CI]	SRMR	*∆χ*^2^	*∆df*
*PsyCap measurement model*									
MMof – one-factor model	517.16**	227	2.28	0.84	0.83	0.08 [0.07–0.09]	0.07		
MMff – second-order, four-factor model	397.64**	221	1.80	0.91	0.90	0.06 [0.05–0.07]	0.06	119.52**	6
*Measurement model*									
MM1 – six factors model (PC, WE, WLB, ES, MH, PF)	501.98**	237	2.12	0.91	0.90	0.07 [0.06–0.08]	0.07		
MM2 – five factors model (PC, WE, WLB, EWB = ES + MH, PF)	654.04**	242	2.70	0.87	0.84	0.09 [0.08–0.10]	0.10	152.06**	5
MM3 – four factors model (PC, WLB, well-being related variables = WE + ES + MH, PF)	1045.05**	246	4.25	0.74	0.70	0.13 [0.12–0.14]	0.12	543.07**	9
MM4 – three factors model (WLB, PF, all other variables)	1233.88**	249	4.96	0.68	0.64	0.14 [0.13–0.15]	0.12	731.90**	12
MM5 – two factors model (PF, all other variables)	1593.37**	251	6.34	0.57	0.51	0.16 [0.16–0.17]	0.14	1091.39**	14
MM6 – single-factor model	1724.47**	252	6.84	0.53	0.47	0.17 [0.16–0.18]	0.15	1222.5**	15
*Structural model*									
SM1 – hypothesized model	166.68**	96	1.73	0.96	0.95	0.06 [0.04–0.07]	0.06		
SM2 – extended work-life model	159.45**	95	1.67	0.97	0.96	0.06 [0.04–0.07]	0.05	7.23**	1
SM3 – extended home-life model	166.29**	95	1.75	0.96	0.95	0.06 [0.05–0.08]	0.06	0.39	1
SM4 – full extended model	158.92**	94	1.69	0.97	0.96	0.06 [0.04–0.07]	0.05	7.76*	2
SM5 – hypothesized model controlling for age	196.67**	109	1.82	0.95	0.94	0.06 [0.05–0.04]	0.06	29.99**	13

^*N* = 217; For MMof the comparison is versus MMff, for MM2, MM3, MM4, MM5, MM6 the comparison is versus MM1, while SM1 is compared to SM2, SM3, SM4, SM5; ^*^p < 0.05, ^*^^*^p < 0.001. PC = psychological capital, WE = work engagement, ES = entrepreneurial satisfaction, WLB = work-life balance, MH = mental health, EWB = entrepreneurial well-being, PF = performance.^

Next, we tested five structural models (SM), where PsyCap predicts venture performance through multiple mediation mechanisms. The first structural model is the hypothesized model (SM1). In SM1, PsyCap predicts entrepreneurial satisfaction both through work engagement and directly and mental health through work-life balance and directly, while entrepreneurial satisfaction and mental health jointly predict business performance (see Figure 1). The second structural model is the extended work-life model (SM2). In this model, in addition to the relationships in SM1, we added a link between work engagement and mental health. Thus, in this model, aspects of the job (i.e., work engagement) positively impact individuals’ work and home roles (i.e., entrepreneurial satisfaction and mental health). Conversely, the third structural model depicts an extended home-life model (SM3). Here, we include a link between work-life balance and entrepreneurial satisfaction next to the hypothesized relationships in SM1. Hence, in SM3, we investigate whether aspects of the home-related role have a positive crossover impact. The fourth structural model is the fully extended mediation model (SM4). This structural model combines the relationships of all the models above. PsyCap predicts entrepreneurial satisfaction and mental health through both primary mediators (i.e., work engagement and work-life balance) and directly. The two EWB components are then linked to business performance. Finally, the fifth structural model (SM5) specifies the same relationships as the hypothesized model (SM1) yet controls for participants’ age, a demographic covariate that correlates with some of the models’ constructs (i.e., work-life balance, mental health, and business performance).d age as a control variable in SM5.

## 3. Results

### 3.1. Descriptive statistics and correlations

The means, standard deviations, Cronbach’s alpha values, and the correlation matrix of the used variables are presented in Table 1. Most correlations between the study variables are statistically significant, except for work-life balance and work engagement and its subscales. Furthermore, the correlations among the dimensions of particular constructs (e.g., PsyCap) are high, thus validating their grouping in higher-order factors. Another noteworthy aspect is the negative correlation between age and tenure in leading current businesses and business performance. Based on the instruments we used, we argue that younger entrepreneurs with lower tenure in leading their business are more prone to develop said business, for example, in terms of employee growth, which may explain these results. Additionally, we calculated the average variance extracted (AVE; Fornell and Larcker, 1981), with the AVE for each construct being reported in Table 1. As can be observed in Table 1, the square root of the AVE extracted by any construct is higher than the correlation between the construct and any other variable, thus demonstrating, from a psychometric perspective, the distinctiveness of each variable included in this study (Fornell and Larcker, 1981; Farrell, 2010). This argument is strengthened by the results of the measurement models, where collapsing variables into super-ordinate factors (e.g., entrepreneurial satisfaction and mental health into EWB; MM2) yields worse fit indices than the original six-factor measurement model (MM1), where each construct is operationalized individually (for details, see Table 2).

### 3.2. Model comparison

Table 2 presents the fit indices for our models. All models yield excellent fit indices. When comparing the alternative models to the hypothesized model (SM1), based on the chi-square difference test, we identified the extended work-life model (SM3) (Δ*χ*^2^ (1) = 7.23, *p* < 0.001) and the full extended mediation model (SM4) as superior (Δ*χ*^2^ (2) = 7.76, *p* < 0.05). The extended home-life model (SM2) did not differ from the hypothesized model (SM1) in a statistically significant manner (Δ*χ*^2^ (1) = 0.39, *p* > 0.05), while SM5, where participants’ age is controlled for, displayed poorer fit indices than the hypothesized model (SM1; Δ*χ*^2^ (13) = 29.99, *p* < 0.001). However, work-life balance did not predict entrepreneurial satisfaction in either of the alternative models employing this relationship (SM3 and SM4). This was also the case for the relationship between work engagement and mental health, specified in the extended work-life model (SM2). In SM5, age was a significant negative predictor of business performance but did not affect any other model relationships. Considering that the hypothesized model (SM1) is the only model with robust fit indices, where all specified relationships are statistically significant, and following Bernerth and Aguinis’s (2016) recommendations, we have come to consider the hypothesized model (SM1; *χ*^2^ (96) = 166.68, *p* < 0.001, CFI = 0.96; TLI = 0.95; RMSEA = 0.06, 90% CI [0.04, 0.07], SRMR = 0.06) as the preferable model.

### 3.3. Hypothesis testing

**Figure 2:**
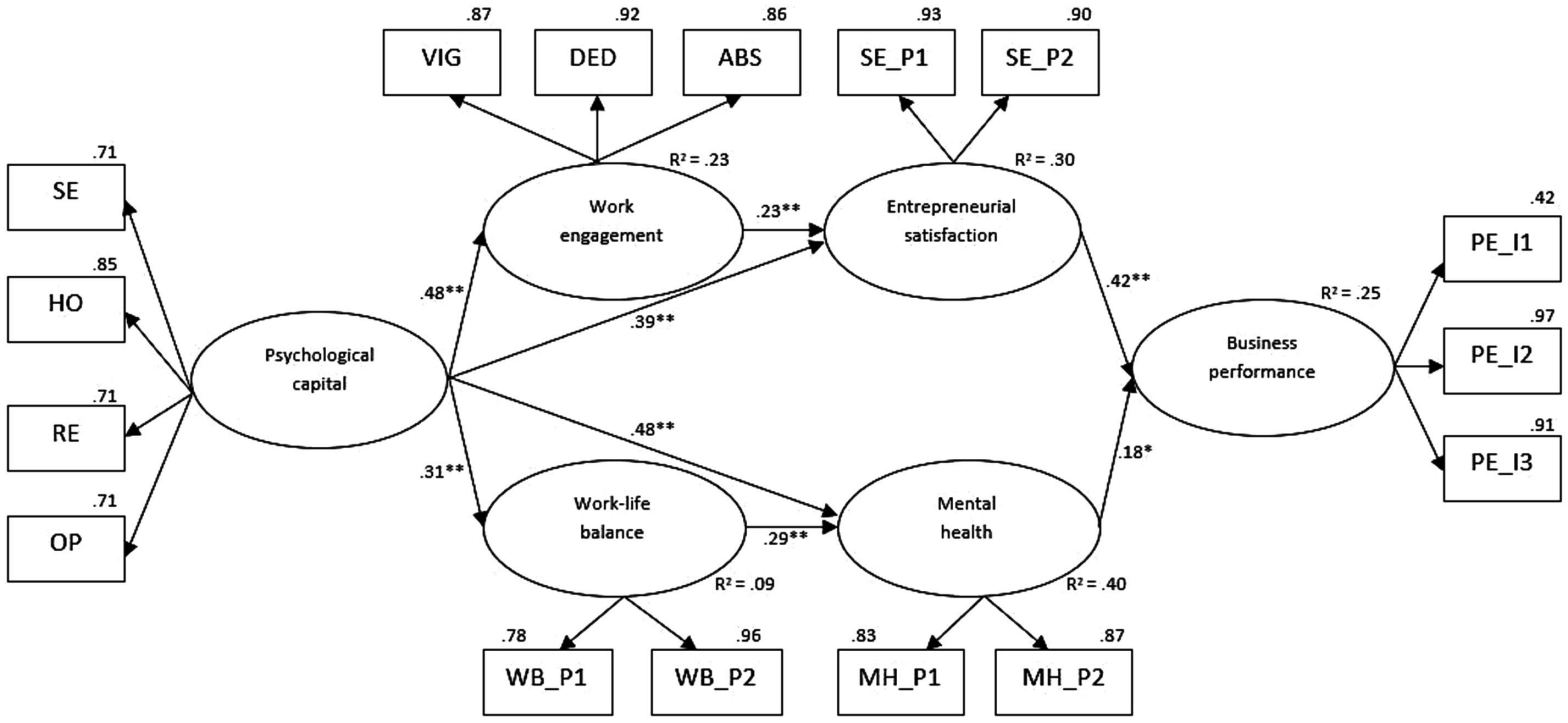
The results of the hypothesized mediation model. The manifest variables used to construct the latent variables reflect the construct’s dimension (for PsyCap and work engagement), parcels (for work-life balance, mental health, and entrepreneurial satisfaction), individual items (for business performance). Standardized regression coefficients are presented in this figure.

Figure 2 displays the results for the hypothesized model (SM1). Concordant with Hypothesis 1a, PsyCap was positively related to entrepreneurs’ work engagement (β = 0.48, *p* < 0.001). Next, work engagement was positively associated with entrepreneurial satisfaction (β = 0.23, *p* < 0.001), favoring Hypothesis 1b. Consistent with Hypothesis 1c, PsyCap also showed a positive link to entrepreneurial satisfaction (β = 0.39, *p* < 0.001). Hypothesis 1d was also supported, with entrepreneurial satisfaction showing a positive relationship with performance (β = 0.42, *p* < 0.001). Also, concordant with Hypothesis 2a, PsyCap was positively related to entrepreneurs’ work-life balance (β = 0.31, *p* < 0.001). Work-life balance showed a positive link to the entrepreneurs’ mental health (β = 0.29, *p* < 0.001), thus favoring Hypothesis 2b. Consistent with Hypothesis 2c, PsyCap predicted entrepreneurs’ mental health (β = 0.48, *p* < 0.001). Finally, Hypothesis 2d was also supported, with entrepreneurs’ mental health yielding a positive association with business performance (β = 0.18, *p* < 0.05). Therefore, the relationship between PsyCap and business performance is mediated through a serial mediation mechanism enabled by work engagement and entrepreneurial satisfaction (indirect effect = 0.05, 95% CI = [0.01–0.07]), while also through a simple mediation mechanism enabled by entrepreneurial satisfaction (indirect effect = 0.16, 95% CI [0.04–0.23]). Furthermore, the relationship between the predictor and the outcome is also mediated through a serial mediation mechanism, namely through work-life balance and mental health (indirect effect = 0.02, 95% CI [0.01–0.03]), and a simple mediation mechanism through mental health (indirect effect = 0.09, 95% CI [0.01–0.13]) (for details, see Table 3).

**Table 3:** Standardized indirect effects with bootstrapped 95% confidence intervals.

Independent variable	Mediator 1	Mediator 2	Dependent variable	Estimate	95% CI
Psychological capital →	Work engagement →	Entrepreneurial satisfaction →	Business performance	0.05*	[0.01–0.07]
Psychological capital →	Entrepreneurial satisfaction →		Business performance	0.16*	[0.04–0.23]
Psychological capital →	Work-life balance →	Mental health →	Business performance	0.02*	[0.01–0.03]
Psychological capital →	Mental health →		Business performance	0.09*	[0.01–0.13]

^*N* = 217, ^*^p < 0.05.^

To conclude, the data supported the hypothesized model and explained 9% of the variance in work-life balance, 23% in the case of work engagement, 30% in the case of entrepreneurial satisfaction, and 40% in the variance in mental health. Finally, our model also accounted for 25% of the variance in business performance.

## 4. Discussion

The primary purpose of this study was to test a comprehensive model, including antecedent (i.e., PsyCap), mediator (i.e., EWB), and outcome (i.e., business performance), exploring whether the cognitive (i.e., entrepreneurial satisfaction) and affective (i.e., mental health) EWB components coexist and jointly predict business performance. This was done by concomitantly investigating the role of business owners and that of an individual outside of work. Furthermore, we sought to identify specific mechanisms related to entrepreneurs experiencing EWB, employing a comprehensive approach consisting of work- (i.e., work engagement) and personal-life-related factors (i.e., work-life balance). Our model, where entrepreneurs’ PsyCap shows an association with business performance through a serial mediation mechanism enabled by work engagement and entrepreneurial satisfaction regarding their work role, and work-life balance and mental health regarding their home role, was fully supported by the collected data, confirming all hypotheses. The model also accounts for one-quarter of the variance in business performance, demonstrating that the model manages to capture highly relevant psychological factors linked to business performance. Furthermore, it shows that while the two EWB components jointly predict business performance, they are distinct factors stemming from different life avenues, with entrepreneurial satisfaction being a stronger predictor than mental health.

These findings support the claim that the entrepreneur is the crucial agent in the entrepreneurial process (Baron, 2007; Gorgievski and Stephan, 2016). Should entrepreneurs experience EWB, this will be reflected in the venture’s performance, which is consistent with previous research investigating the entrepreneurial well-being domain (Wincent et al., 2008; Stephan, 2018; Wiklund et al., 2019; Kleine and Schmitt, 2021). Our findings may be explained through the lens of COR theory (Hobfoll, 2011), which states that individuals who have an abundant resource-reservoir (i.e., PsyCap, EWB) can successfully invest their cognitive and emotional resources into actual behaviors (e.g., meeting with prospective clients, devising new products), thus being able to acquire resources that enable their business to grow and thrive.

Regarding our hypotheses, PsyCap showed an association with entrepreneurial satisfaction directly and through a mediation mechanism enabled by work engagement, with the former positively predicting business performance. Hence, when looking at the work-life avenue of entrepreneurs, it seems that the success-oriented mindset represented by PsyCap is indeed a “fuel” that enables entrepreneurs to experience EWB through the “combustion method” of work engagement, which then reflects on entrepreneurial satisfaction and, indirectly, business performance. First, being equipped with the positive, agentic cognitive, and emotional resources of PsyCap’s components, such as being able to set specific goals and trusting one’s ability to meet those challenges or having an optimistic view about one’s business, enables entrepreneurs to experience a high-energy activating sense of enjoyment while being immersed in their work. These findings support the claim of Van den Heuvel et al. (2010), who argued that high PsyCap levels would allow individuals to experience a sense of work engagement, even regarding activities that are not necessarily perceived as pleasant or in activities dominated by uncertainty, a hallmark of entrepreneurship (Stephan, 2018). Next, the relationship mentioned above predicts entrepreneurial satisfaction, where entrepreneurs are content with their entrepreneurial lives. Thus, our findings identify a positive mediator through which PsyCap impacts entrepreneurial satisfaction, expanding the findings of Baron et al. (2016), who found a similar association between the two concepts, with perceived stress as a mediator. We also found entrepreneurial satisfaction to predict business performance positively, which aligns with other researchers’ findings (Dijkhuizen et al., 2018; Stephan, 2018). Entrepreneurs who are satisfied with their life as entrepreneurs, indeed, have more psychological resources at their disposal (Hobfoll, 2011) and invest these to develop their businesses (Wu, 2007).

Similarly, regarding the personal-life avenue of entrepreneurs, PsyCap also showed a link to mental health both directly and through a mediation mechanism enabled by work-life balance, with mental health also positively predicting business performance. Hence, PsyCap is a relevant resource when exploring entrepreneurs’ work-life avenue and their private lives. Detailing our results, PsyCap shows an association with entrepreneurs experiencing work-life balance, which expands the findings of Luthans et al. (2013) by linking work PsyCap to personal-life outcomes and those of Siu (2013) by validating the above relationship on a different occupational category (i.e., entrepreneurs). Therefore, entrepreneurs with high PsyCap levels can find the time to engage in leisure activities with others due to their ability to tackle work-related issues more efficiently (i.e., planning and self-efficacy). This is of utmost importance, considering that entrepreneurs need to satisfy their need for relatedness through activities outside their work-life (Stephan, 2018; Shir et al., 2019), which represents a prerequisite for EWB (Ryan and Deci, 2017; Wiklund et al., 2019). Hence, finding a balance between work and home roles enables not only employees to exhibit good mental health (Haar et al., 2014) but entrepreneurs as well. Next, PsyCap was also directly linked to our sample’s mental health, confirming that this personal resource allows entrepreneurs to experience more positive and less negative feelings in their lives. Setting challenging goals, recovering after setbacks, and having an optimistic worldview enables entrepreneurs to surmount everyday stress or negative ruminations, probably by engaging in resource-replenishing activities (e.g., a night out, practicing sport; Kinnunen et al., 2011; Rodríguez-Muñoz et al., 2014). Thus, PsyCap allows individuals to protect their mental health, acting as a buffer in experiencing stress or other poor mental health triggers. These results expand the positive association between PsyCap and mental health, previously identified on nurses’ samples (Estiri et al., 2016) or soldiers (Krasikova et al., 2015).

Importantly, our employed predictor (i.e., PsyCap) and the explanatory mediating mechanism (i.e., work-life balance) can explain almost half of the mental health variance. This indicates that although we relied upon a parsimonious approach, it is a comprehensive and efficient model that accurately identifies triggers of EWB. Finally, mental health was found to be a precursor of business performance, a result that is consistent with previous findings (Stephan, 2018). Entrepreneurs, being able to glean resources due to not experiencing poor mental health, can invest them in work-related activities, reflecting on their business performance.

### 4.1. Limitations and future directions

As with any research, our study does come with several limitations. First, we relied on a cross-sectional design, which inhibits the identification of causal mechanisms. However, our results provide an exploratory model upon which a future longitudinal design may be employed to generate a more solid causal-like model. Furthermore, our results build upon earlier longitudinal findings, such as the multi-wave studies of Siu (2013), who linked PsyCap to work-life balance, and those of Laguna et al. (2017), who linked self-efficacy to work engagement. These findings sustain our expectation that the proposed model would pass the scrutiny of a longitudinal design. Future randomized controlled trials would then provide experimental evidence of our assumption that PsyCap positively impacts business performance through increased EWB. For instance, we suggest a three-pronged approach to developing entrepreneurs’ PsyCap and subsequent EWB that includes their work and home roles. Following the entrepreneurial PsyCap intervention developed by Zeng et al. (2022), practitioners could develop and provide entrepreneurs with structured reading materials (e.g., inspiring stories of successful entrepreneurs) to enhance their PsyCap and work engagement. Similarly, entrepreneurs can be provided with information regarding how balancing their roles can result in enhanced performance and how they can use their autonomy to enact behaviors that sustain their involvement in both roles. Furthermore, they can be encouraged to identify those idiosyncratic strategies they rely upon to optimize their energy levels and include relevant stakeholders from their personal lives in those activities (Tisu and Vîrgă, 2022). This process can be bolstered through the usage of implementation intentions – if-then plans that can help translate objectives into action (e.g., “If I will visit a museum to find inspiration, then I will ask my spouse to join me”; Gollwitzer and Sheeran, 2006). Additionally, the reading materials and formulation of implementation intentions could be coupled with short mindfulness sessions, which can help entrepreneurs detach from work-related stress, thus increasing their PsyCap levels (for details, see Zeng et al., 2022), reflecting on their EWB.

Secondly, while our model proposes unidirectional associations between PsyCap, EWB, and performance, both COR (Hobfoll, 2011) and JD-R (Bakker and Demerouti, 2017; Bakker and De Vries, 2021) theories advocate for mutual reinforcing effects between an individual’s personal resources (e.g., PsyCap) and work-related outcomes (e.g., performance). These theoretical developments suggest that reciprocal effects between PsyCap, EWB, and business performance could occur. In other words, it is also plausible that business performance can help foster EWB, which, in turn, may replenish entrepreneurs’ PsyCap levels. For instance, if a business attains good performance indicators due to entrepreneurs’ initial success-oriented mindset (i.e., PsyCap), this should allow entrepreneurs to experience enhanced satisfaction and, probably, reduced stress levels (i.e., mental health) in return. As such, they may find it easier to engage in non-work-related activities with family and friends (i.e., work-life balance), thus replenishing their psychological resources pool (i.e., PsyCap). Indeed, Tisu and Vîrgă (2022) capture such reciprocal relationships in a two-wave study on Romanian entrepreneurs, showing that entrepreneurs who optimize their energy levels through proactive behaviors (i.e., proactive vitality management) can better handle the work-home mélange, a prerequisite of work-life balance. In turn, enriching their home life through aspects of their work enables entrepreneurs to sustain their initial proactive vitality management behaviors in a reciprocal gain spiral (Hobfoll, 2011; Tisu and Vîrgă, 2022). Thus, we encourage researchers to further explore dynamic relationships between entrepreneurs’ resources, proactive behaviors, and business outcomes to understand better how they influence and sustain each other.

Third, our data were collected using self-report questionnaires, which may affect the data’s trustworthiness due to common method bias occurrence (Podsakoff et al., 2012). However, the common-method model (MM6) displayed poor fit indices, while the six-factor model (MM1) had good fit indices, thus indicating a low chance of common method bias on our sample. Also, we measured business performance in terms of venture growth. While this approach is established in the literature (Dijkhuizen et al., 2016a,b), a lack of development does not necessarily imply an absence of performance (Wiklund et al., 2019). Therefore, future studies should seek to employ more diverse means of data collection, such as objective indicators of business performance, and expand the concept of performance beyond venture growth. Also, two of the PsyCap subscales’ internal consistency (optimism and resilience) were slightly below the 0.70 threshold. However, as Schmitt (1996) noted, the cut-off value of 0.70 is rather indicative than definitive. Thus, our findings should not be affected by this issue.

Fourth, considering we relied upon a convenience sample, it may be possible that only entrepreneurs who already exhibit a certain level of PsyCap, thus not experiencing high levels of work-related strain, engaged in responding to our questionnaire, an issue which may affect the generalization of our findings (Stephan, 2018; Wiklund et al., 2019). Therefore, we recommend that future studies rely on more robust sampling methods, such as including and differentiating between opportunity and necessity entrepreneurs (Gorgievski and Stephan, 2016) or individual and team entrepreneurs. It should be noted that the sampling procedure applied here may have led to a relatively homogeneous sample compared to the general population of entrepreneurs. This will likely result in a conservative estimation of the study variables’ associations due to restriction-of-range effects (i.e., although some bias may occur, our findings will not be overestimated). Considering our sample’s possible lack of heterogeneity, we expect future studies to employ more solid sampling methods to identify more systematic variance and less error, thus obtaining stronger associations.

### 4.2. Theoretical and practical implications

This study contributes to the development of entrepreneurship literature by advancing our understanding regarding the emergence of EWB, how it may be fostered, and its link to business performance. From a theoretical perspective, this study establishes PsyCap as an antecedent of EWB and identifies mediators that explain this relationship. Thus, it answers the call of Obschonka and Stuetzer (2017), who encourage researchers to identify malleable psychological constructs that can be developed through interventions to enhance entrepreneurial performance and, thus, business performance. Also, it heeds the call of Wiklund et al. (2019), who argue that research should focus on uncovering mediators related to the emergence of EWB. Based on our results, PsyCap represents a developable psychological resource that equips entrepreneurs with a success-oriented mindset that allows them to be both satisfied with their lives as entrepreneurs and exhibit good mental health, the two distinct components of EWB captured in this study. On the one hand, drawing their energies from high PsyCap levels allows entrepreneurs to perceive more of their activities as engaging, reflecting on their entrepreneurial satisfaction, thus exhibiting the belief that they are content with their lives as entrepreneurs. On the other hand, it enables them to find a balance between work- and non-work-related activities. Managing to handle both roles effectively allows entrepreneurs to engage in resource-replenishing activities in their home role (Kinnunen et al., 2011), which can reduce stress levels, generate positive feelings, and protect their mental health. Considering our sample comprises both entrepreneurs who have only recently started their businesses and seasoned entrepreneurs, finding PsyCap as an antecedent of EWB highlights its usefulness in various stages of the business’s development. However, PsyCap is only one personal resource. Future studies should aim to identify and integrate other such malleable constructs, like meaning-making (Van den Heuvel et al., 2010), that can help entrepreneurs thrive in their roles by experiencing EWB. Tisu and Vîrgă (2021) have taken steps in this direction, showing that meaning-making moderates the link between entrepreneurial development opportunities and entrepreneurial performance. Furthermore, Wiklund et al. (2019) propose a wide array of EWB indicators, such as personal growth, meaning, mastery, or positive relations, that can be incorporated into future models.

Second, the present investigation captures the essence of EWB by concomitantly exploring both entrepreneurial satisfaction and entrepreneurs’ mental health, showing they may coexist when integrating entrepreneurs’ work and home roles. Importantly, we find that the cognitive (i.e., entrepreneurial satisfaction) and affective (i.e., mental health) components are distinct and have different sources that fuel their emergence. This finding aligns with Pavot and Diener’s (2008) proposition, distinguishing between affective and cognitive dimensions of well-being. Incorporating other relevant EWB constructs (e.g., personal growth, meaning; see Wiklund et al., 2019) into future models could establish EWB as a super-ordinate factor. Also, this study goes beyond eudaimonic indicators of well-being (i.e., entrepreneurial satisfaction) and includes activating mechanisms (i.e., work engagement) reflecting the hedonic component of well-being (Wiklund et al., 2019). Perceiving most of their daily activities as thrilling and pleasant (i.e., work engagement) triggers, as an aggregate, a sense of satisfaction with their lives as entrepreneurs. Thus, it provides additional evidence for the COR theory’s assumption regarding the emergence of resource caravans (Hobfoll, 2011). Entrepreneurs who experience work engagement also exhibit satisfaction with their lives as entrepreneurs. Importantly, COR theory also stipulates that positive gain spirals may occur, with resources reinforcing each other over time. Thus, while PsyCap appears to fuel work engagement and satisfaction, it is possible that the latter two may also replenish the invested PsyCap over time. Laguna et al. (2017) have investigated such dynamic relationships between entrepreneurs’ self-efficacy and work engagement, finding no such links. However, in their study, the authors only employed one of the four PsyCap components, which may prove insufficient to capture reinforcing relationships due to inadequate statistical power. Therefore, by validating PsyCap as an antecedent of EWB, we provide additional evidence that researchers should capture this construct as a relevant personal resource in entrepreneurship to achieve more realistic modeling, possibly capturing existing positive gain spirals.

Third, our findings’ crucial implication is that entrepreneurs should not sacrifice their personal lives for their work lives because this will not benefit their businesses. Nowadays, it is common for some entrepreneurs to focus all their energies on work, neglecting their personal lives (Ezzedeen and Zikic, 2017; Adisa et al., 2019). However, this will burden their optimal psychological functioning because their need for relatedness is frustrated. Indeed, Shir et al.’s (2019) findings indicate that engaging in entrepreneurship does not satisfy this basic psychological need. Due to the lonely nature of entrepreneurship, entrepreneurs will lack social support, a vital element for good mental health (Stephan, 2018). As this study demonstrates, to experience EWB and have sufficient resources to help their business grow, entrepreneurs must also experience good mental health. Therefore, it is paramount that entrepreneurs devote some of their time to non-work-related activities as well, which can reduce stress levels, experience more positive affect, and safeguard their optimal psychological functioning. Only then can they fully experience EWB, a precursor of business performance.

While it may be argued that relatedness may be attained through business-related activities, such as meeting with clients or fellow entrepreneurs, our results suggest the contrary. The alternative models tested in this paper, which specified crossover relationships from one life domain to the other, did not contain any such significant relationships. Work engagement was not an antecedent of mental health, nor was work-life balance a predictor of entrepreneurial satisfaction. Therefore, engaging in activities in the work role is not sufficient for entrepreneurs to experience good mental health. We acknowledge that it may prove challenging to convince entrepreneurs to devote time to activities outside of work. However, this must be done for them to fully experience EWB, thus accumulating sufficient resources to help their business thrive.

From a practical perspective, we have provided practitioners with a developable personal resource that can be fostered among entrepreneurs to enhance their business performance. As the meta-analysis of Lupșa et al. (2020) indicates, interventions based on PsyCap development yield fruitful results. They are also time- and cost-efficient and may even be administered online (Lupșa et al., 2020). Practitioners can organize sessions with entrepreneurs to train them in setting relevant business-related goals (hope), develop their confidence in attaining these objectives (self-efficacy), and help them put things in perspective after potential setbacks, thus generating resilience. These aspects also enable entrepreneurs to develop an optimistic outlook on their activities. This should allow them to find more of their activities engaging, reflecting on entrepreneurial satisfaction. Importantly, these activities should not be focused exclusively on their work role but also contain elements regarding how to organize their home-life, too. Entrepreneurs can be presented with the benefits of engaging in non-work-related activities to replenish their resource reservoir and reduce strain. Next, they can be helped to generate a daily or weekly schedule that involves spending time with family or friends, practicing sports, or doing volunteer work to satisfy the need for relatedness, gain social support, and experience optimal mental health, a prerequisite for optimal business functioning. Should entrepreneurs encounter difficulties in participating in coaching sessions, they could still engage in short online interventions to develop and sustain their PsyCap and EWB, such as the one advanced by Zeng et al. (2022), which we described in the previous section. Furthermore, entrepreneurs may be taught how to use their autonomy to detach from work and rely on implementation intentions (if-then plans that facilitate goal attainment; Gollwitzer and Sheeran, 2006) to translate these objectives into action. For instance, entrepreneurs could devise if-then plans to ensure that they celebrate attaining various goals (e.g., “If I manage to sell *n* products in a day, then I will go out on that evening with friends/employees to celebrate”). Existing interventions based on implementation intentions as a self-regulatory mechanism yield fruitful results in various domains (for synthesis, see Bieleke et al., 2021). We expect entrepreneurs to make no exception.

## 5. Conclusion

This study demonstrates that PsyCap, a malleable personal resource, is linked to business performance via EWB. Also, EWB emerges as a consequence of optimal functioning in the (1) work role, through work engagement that leads to entrepreneurial satisfaction, and (2) home role, where work-life balance predicts good mental health. Thus, our findings suggest a need for a paradigm shift. It is time to no longer view entrepreneurs’ work and home roles as identical, considering that one covers the other, at least not regarding entrepreneurs’ psychological well-being. Currently, that is the case because many entrepreneurs tend to sacrifice the latter, assuming only a work-related role in their lives. This needs to change, with entrepreneurs setting clear boundaries between their roles and engaging in both. Based on this study’s results, entrepreneurs’ work and home roles are separate components that lead to domain-specific outcomes (i.e., entrepreneurial satisfaction and mental health) linked to business performance. As such, the two life avenues should be addressed concomitantly yet as distinct components, both in theory and practice, to accurately grasp the emergence of EWB and its relation to business performance.

## Data availability statement

The raw data supporting the conclusions of this article will be made available by the authors, without undue reservation.

## Ethics statement

Ethical approval was not required for the studies involving humans because it is a survey research and does not involve manipulation. The studies were conducted in accordance with the local legislation and institutional requirements. The participants provided their written informed consent to participate in this study.

## Author contributions

LT and DV conceived the presented idea. LT developed the theory and performed the computations. DV and TT verified the analytical methods and guided the theoretical argumentation process. All authors discussed the results and contributed to the final manuscript.
