# Supplementary material for: Entrepreneurial well-being and performance: antecedents and mediators
Source: Front Psychol. 2023 Oct 19;14:1112397. doi: 10.3389/fpsyg.2023.1112397 (PMC10620712; doi:10.3389/fpsyg.2023.1112397)
Supplement: Supplementary file 1 [file Table_1.docx]

**Supplementary Table 1**. Item wording for each variable, the parcel that variables were included in, results of confirmatory factor analysis for each latent variable, and descriptive statistics for each item

| **Construct** | **Parcel** | **CFA results** | **Item** | **M** | **SD** | **SK** | **KU** | **FL** |
| --- | --- | --- | --- | --- | --- | --- | --- | --- |
| Psychological capital | Self-efficacy | χ2(6) = 17.09*  CFI = .98  TLI = .95  RMSEA = .08  SRMR = .04 | I feel confident analyzing a long-term problem to find a solution in my work as an entrepreneur. | 4.94 | .96 | -1.09 | 1.79 | .61 |
|  |  |  | I feel confident in representing my work area in meetings with stakeholders (e.g., clients, investors). | 5.08 | .93 | -1.02 | .95 | .75 |
|  |  |  | I feel confident leading discussions about my company's strategy. | 5.00 | 1.06 | -1.18 | 1.12 | .77 |
|  |  |  | I feel confident helping to set targets/goals in my work as an entrepreneur. | 5.34 | .78 | -.98 | .35 | .72 |
|  |  |  | I feel confident contacting people outside the company (e.g., suppliers, customers) to discuss business-related problems. | 5.23 | .84 | -1.06 | 1.12 | .68 |
|  |  |  | I feel confident presenting information about my business to stakeholders (e.g., clients, investors). | 5.33 | .87 | -1.48 | 2.54 | .60 |
|  |  |  |  |  |  |  |  |  |
|  | Hope | χ2(6) = 12.34  CFI = .98  TLI = .97  RMSEA = .06  SRMR = .04 | If I should find myself in a jam in my work as an entrepreneur, I could think of many ways to get out of it. | 5.07 | 1.08 | -1.07 | 5.04 | .63 |
|  |  |  | At the present time, I am energetically pursuing my work goals as an entrepreneur. | 5.18 | .78 | -1.01 | 1.86 | .61 |
|  |  |  | There are lots of ways around any problem in my activity as an entrepreneur. | 5.16 | .87 | -1.03 | 1.04 | .61 |
|  |  |  | Right now I see myself as being pretty successful in my work as an entrepreneur. | 4.73 | 1.08 | -.82 | .26 | .55 |
|  |  |  | I can think of many ways to reach my current work goals as an entrepreneur. | 5.07 | .84 | -.78 | .47 | .81 |
|  |  |  | At this time, I am meeting the work goals that I have set for myself as an entrepreneur. | 3.89 | 1.23 | -.52 | -.35 | .89 |
|  |  |  |  |  |  |  |  |  |
|  | Resilience | χ2(6) = 14.91*  CFI = .96  TLI = .90  RMSEA = .08  SRMR = .05 | When I have a setback in my work as an entrepreneur, I have trouble recovering from it and moving on. (R) | 4.05 | 1.25 | -.61 | -.42 | .57 |
|  |  |  | I usually manage difficulties one way or another in my work as an entrepreneur. | 4.82 | .84 | -1.13 | 2.71 | .51 |
|  |  |  | I can be "on my own" so to speak in my work as an entrepreneur, if I have to. | 5.12 | .96 | -1.39 | 2.23 | .65 |
|  |  |  | I usually take stressful things in stride in my work as an entrepreneur. | 4.44 | 1.05 | -.67 | .52 | .74 |
|  |  |  | I can get through difficult times in my work as an entrepreneur because I've experienced difficulty before. | 4.78 | .92 | -.56 | .07 | .51 |
|  |  |  | I feel I can handle many things at a time in my work as an entrepreneur. | 5.08 | .80 | -.88 | 1.04 | .82 |
|  |  |  |  |  |  |  |  |  |
|  | Optimism | χ2(5) = 11.80*  CFI = .97  TLI = .91  RMSEA = .08  SRMR = .04 | When things are uncertain for me in my work as an entrepreneur, I usually expect the best. | 4.32 | 1.11 | -.69 | .38 | .63 |
|  |  |  | If something can go wrong for me as an entrepreneur it will. (R) | 3.09 | 1.23 | -.52 | .10 | .50 |
|  |  |  | I always look on the bright side of things regarding my work as an entrepreneur. | 4.84 | .86 | -.54 | .21 | .74 |
|  |  |  | I’m optimistic about what will happen to me in the future as it pertains to my work as an entrepreneur. | 5.03 | .91 | -1.12 | 1.96 | .66 |
|  |  |  | In my work as an entrepreneur, things never work out the way I want them to. (R) | 4.80 | 1.23 | -1.11 | .77 | .49 |
|  |  |  | I approach my activity as an entrepreneur as if every cloud has a silver lining. | 4.97 | .80 | -.64 | .83 | .64 |
|  |  |  |  |  |  |  |  |  |
| Work engagement | Vigor | χ2(22) = 50.51*  CFI = .97  TLI = .95  RMSEA = .08  SRMR = .04 | At my work, I feel bursting with energy. | 5.00 | 1.52 | -.69 | -.19 | .61 |
|  |  |  | At my job, I feel strong and vigorous. | 5.48 | 1.38 | -.78 | -.26 | .79 |
|  |  |  | When I get up in the morning, I feel like going to work. | 5.75 | 1.42 | -1.54 | 1.99 | .65 |
|  | Dedication |  | I am enthusiastic about my job. | 5.75 | 1.32 | -1.22 | 1.08 | .82 |
|  |  |  | I find the work that I do full of meaning and purpose. | 5.87 | 1.42 | -1.34 | 1.25 | .83 |
|  |  |  | I am proud of the work that I do. | 6.23 | 1.14 | -1.71 | 2.56 | .73 |
|  | Absorption |  | I feel happy when I am working intensely. | 5.71 | 1.41 | -1.29 | 1.52 | .75 |
|  |  |  | I am immersed in my job. | 5.71 | 1.43 | -1.32 | 1.49 | .69 |
|  |  |  | Time flies when I am working. | 5.32 | 1.75 | -.98 | .07 | .71 |
|  |  |  |  |  |  |  |  |  |
| Entrepreneurial satisfaction | SE_P2 | χ2(2) = 1.46  CFI = 1.00  TLI = 1.00  RMSEA = .01  SRMR = .01 | In most ways, my life as an entrepreneur is close to my ideal. | 5.34 | 1.23 | -.79 | .41 | .81 |
|  | SE_P2 |  | The conditions of my life as an entrepreneur are excellent. | 5.46 | 1.12 | -.96 | .95 | .86 |
|  | SE_P1 |  | I am satisfied with my life as an entrepreneur. | 5.61 | 1.10 | -1.06 | 1.18 | .91 |
|  | SE_P2 |  | So far I have gotten the important things I want in my life as an entrepreneur. | 5.39 | 1.16 | -.85 | .45 | .85 |
|  | SE_P1 |  | If I could live my life as an entrepreneur over, I would change almost nothing. | 5.06 | 1.41 | -.75 | -.01 | .76 |
|  |  |  |  |  |  |  |  |  |
| Work-life balance | WB_P2 | χ2(2) = 2.33  CFI = 1.00  TLI = 1.00  RMSEA = .03  SRMR = .02 | I currently have a good balance between the time I spend at work and the time I have available for non-work activities | 3.59 | 1.04 | -.57 | -.38 | .83 |
|  | WB_P1 |  | I have difficulty balancing my work and non-work activities. | 3.17 | 1.12 | -.20 | -.90 | .62 |
|  | WB_P1 |  | I feel that the balance between my work demands and non-work activities is currently about right. | 3.51 | .95 | -.34 | -.02 | .86 |
|  | WB_P2 |  | Overall, I believe that my work and non-work life are balanced. | 3.72 | .95 | -.70 | .29 | .85 |
|  |  |  |  |  |  |  |  |  |
| Mental health | MH_P2 | χ2(2) = 3.17  CFI = 1.00  TLI = .98  RMSEA = .05  SRMR = .02 | How much of the time, during the last month, have you been a very nervous person? (R) | 4.05 | 1.26 | -.76 | .03 | .66 |
|  | MH_P2 |  | How much of the time, during the last month, have you felt calm and peaceful? | 4.20 | 1.12 | -.49 | -.29 | .68 |
|  | MH_P1 |  | How much of the time, during the last month, have you felt downhearted and blue? (R) | 4.72 | 1.10 | -1.18 | 1.39 | .89 |
|  | MH_P1 |  | How much of the time, during the last month, have you been a happy person? | 4.40 | .98 | -.82 | .34 | .62 |
|  | MH_P2 |  | How much of the time, during the last month, have you . felt so down in the dumps that nothing could cheer you up? (R) | 5.16 | 1.24 | -1.98 | 3.66 | .71 |
|  |  |  |  |  |  |  |  |  |
| Business performance | PE_I1 | χ2(0) = 0.00  CFI = 1.00  TLI = 1.00  RMSEA = .00  SRMR = .00 | How did the number of employees in your company change over the past twelve months? | 3.12 | .79 | .03 | 1.62 | .41 |
|  | PE_I2 |  | How did the profit margin of the company change over the past twelve months? | 3.50 | .94 | -.43 | -.26 | .99 |
|  | PE_I3 |  | How did the total turnover of the company change over the past twelve months? | 3.46 | .97 | -.59 | -.10 | .88 |

*^Note^*^. M = mean, SD = standard deviation, SK = skewness, KU = kurtosis, FL = standardized factor loadings. Residuals of items with similar wordings comprising a construct were allowed to correlate. * p < .05^
